# A Wireless Sensor Network for Growth Environment Measurement and Multi-Band Optical Sensing to Diagnose Tree Vigor

**DOI:** 10.3390/s17050966

**Published:** 2017-04-27

**Authors:** Shinichi Kameoka, Shuhei Isoda, Atsushi Hashimoto, Ryoei Ito, Satoru Miyamoto, Genki Wada, Naoki Watanabe, Takashi Yamakami, Ken Suzuki, Takaharu Kameoka

**Affiliations:** 1 Graduate School of Bioresources, Mie University, 1577 Kurimamachiya-cho, Tsu City 514-8507, Mie, Japan; 515d201@m.mie-u.ac.jp (S.K.); 107isoda@gmail.com (S.I.); hasimoto@bio.mie-u.ac.jp (A.H); itou-r@bio.mie-u.ac.jp (R.I.); 2 Sumitomo Precision Products Co., Ltd., 1-10 Fuso-cho, Amagasaki City 660-0891, Hyogo, Japan; miyamo-s@spp.co.jp; 3 Tomi no Oka Winery, Suntory Wine International Limited, 2786, Ohnuta Kai-shi 400-0103, Yamanashi, Japan; genki_wada@suntory.co.jp (G.W.); naoki_watanabe@suntory.co.jp (N.W.); 4 Department of Agriculture, Kisyu Agricultural Extension Center, Mie Prefectural Government, 371 Idomachi, Kumano City 519-4300, Mie, Japan; yamakt04@pref.mie.jp (T.Y.); suzukk07@pref.mie.jp (K.S.)

**Keywords:** wireless sensor network (WSN), Wi-SUN, vine, mandarin orange, thermal image, fluorescent measurement, X-ray fluorescence spectroscopy

## Abstract

We have tried to develop the guidance system for farmers to cultivate using various phenological indices. As the sensing part of this system, we deployed a new Wireless Sensor Network (WSN). This system uses the 920 MHz radio wave based on the Wireless Smart Utility Network that enables long-range wireless communication. In addition, the data acquired by the WSN were standardized for the advanced web service interoperability. By using these standardized data, we can create a web service that offers various kinds of phenological indices as secondary information to the farmers in the field. We have also established the field management system using thermal image, fluorescent and X-ray fluorescent methods, which enable the nondestructive, chemical-free, simple, and rapid measurement of fruits or trees. We can get the information about the transpiration of plants through a thermal image. The fluorescence sensor gives us information, such as nitrate balance index (NBI), that shows the nitrate balance inside the leaf, chlorophyll content, flavonol content and anthocyanin content. These methods allow one to quickly check the health of trees and find ways to improve the tree vigor of weak ones. Furthermore, the fluorescent x-ray sensor has the possibility to quantify the loss of minerals necessary for fruit growth.

## 1. Introduction

Agricultural plants are extremely sensitive to climate change. Higher temperatures eventually reduce yields of desirable crops, while encouraging weed and pest proliferation. Changes in precipitation patterns increase the likelihood of short-run crop failures and long-run production declines. Today, the necessity of support of cultivation has been increasing with the escalation of issues such as the decrease in the number of people engaged in agriculture and the aging of this population. 

The development of science-based agriculture is desired in order to adapt to the changing climate and to promote environmentally friendly smart agriculture with energy saving strategies. To that end, two kinds of measurements are indispensable; the first one is establishing a periodic acquisition system of meteorological and soil information at the field and creating cultivation indices by using this information; the second one is establishing the method for examining the tree vigor or balance of nutrition contents in the plant. 

In regard to the first kind of measurement, a Wireless Sensor Network (WSN) is a methodology for acquiring growing environmental information. The WSN is a wireless network of small, low-cost sensors used for monitoring the physical environment at remote locations [1]. Therefore, since 2009, we have been using WSN in a mandarin orange orchard and a vineyard to promote smart cultivation management practices [2,3,4]. Based on these two kinds of fruit-growing examples, we have obtained some knowledge related to the issues of a field sensor network as well as the installation of weather stations and soil moisture sensors. There were also some problems; it takes too much time to restore the WSN system because the sensors and weather station were not homemade; the communication range was limited due to the frequency of radiowaves and the tipping bucket rain gauge needs regular maintenance. 

In terms of the second objective, quality evaluation and control of agricultural products are very important to provide consistently high quality for the cultivation and postharvest management and marketing. Valuable information on plant nutrition needs to be addressed not only in the contents, but also in their balance in plant organs over the entire period of plant growth and the postharvest process. Understanding the change in the balance of elements at the level of field cultivation is another important factor in fruit cultivation, and acquiring the correct information on the balance of elements enables control of the amount of fertilizer and the plant’s environment. Focusing on the relation between recent unstable climate and crops, it is also very important at the level of field cultivation because external environmental factors, such as abnormal climate and air pollution, produce a large change in the balance of the nutritional state in a plant, leading to a decrease in its yield.

So far, we have been developing integrated investigations on the multiband optical sensing of metabolites, biological systems and foodstuffs by using color imaging, and on the applications of such sensing techniques to the measurements of plants and agricultural materials at the field using infrared (IR) spectroscopy, thermal imaging and X-ray fluorescence (XRF) spectroscopy because optical sensing enables the simple, non-destructive, simultaneous, chemical-free, and rapid measurement of plants [4,5,6,7,8]. Especially, element measurement in the leaf (such as K, Ca, P and S) by using XRF spectroscopy and nitrogen measurement in the leaf by using mid-infrared (MIR) spectroscopy show high possibility for quantitative measurement [4,7,8]. In addition, measurement of the leaf temperature by using thermography camera is implemented [5,6]. In recent years, handy types of XRF sensors and fluorescence sensors for pigment analysis have been developed and we could apply these sensors in the field. Therefore, by using these portable sensors, we could achieve the non-disruptive and real-time measurement of the elements and pigments present in the leaves. 

By the way, in Japan, the research for changing from agricultural ICT to agricultural IoT are undertaken as a project supported by the Ministry of Agriculture, Forestry and Fisheries. Agricultural IoT system refers to the whole system that includes the WSN in the field, data cloud containing growth environmental information and a web application service for farmers [9]. By integrating the growth environmental information and tree vigor information in the data cloud, it is possible to provide a more useful and effective service for farmers.

In this study, we developed a revised WSN system at the Tomi-no-oka vineyard toward the next generation of WSN for cultivation management based on several basic concepts. The first one is a locally produced scalable WSN with an affinity similar to the previously used eKo (Crossbow Technology Inc., Milpitas, CA, USA) system. The second one is a locally produced sensor with good support as a general rule. For the third monitoring items in farms are determined from the view point of phenology, plant physiology and synecology. Lastly, the WSN is correctly placed based on the Interoperable Agricultural Information Platform structure. In this sense, some cultivation indices for wine grape are established and used in Europe; we used a vineyard for wine that can verify the validity of these indices. This paper also focuses on the vigor measurement of mandarin orange leaves by using thermal images, fluorescence and fluorescence X-ray methods in the field. Since there is a lot of available information about nutrient measurement of mandarin orange tree in Japan, a mandarin orange field was used in this experiment.

## 2. Object Fields and Methods 

### 2.1. Object Fields

We have now been conducting demonstration experiments of agricultural WSN applications for more than three years using two farms. One is a mandarin orange grove (north latitude: 33.8634418°, east longitude: 136.05652822°), located at the Kanayama pilot farm in Kumano City, Mie Prefecture, Japan and the other is a vineyard (north latitude: 35.7103912885258°, east longitude: 138.5118197255086°), the Suntory Tomi-no-oka Winery in Kai city, Yamanashi Prefecture, Japan.

### 2.2. Growing Environmental Measurement by Using WSN

An eKo wireless sensor network has been used at the vineyard in the Suntory Tomi-no-oka Winery for climate and soil moisture measurement. In order to update the WSN for the next generation WSN, there are four points of modification from our previous study [4]. 

First, because of its maintenance, the eKo WSN should be replaced with another system that is domestically produced. Although the former eKo WSN system had been working effectively for more than five years, the plastic and battery inside the eKo deteriorate due to photoreactions and we should address this issue. In addition, once the system was broken, it took too much time since the eKo could not be repaired in Japan. In order to maintain the durability of the WSN system for a long time, a domestic system is favored in the viewpoint of quick and smooth restoration. Thus, the eKo WSN was replaced with a new system (Sumitomo Precision Products Co., Ltd., Amagasaki, Japan).

Secondly, the sensors used in the new WSN should be domestically made or maintenance free from the point of view of operation and maintenance. The soil water potential sensors were homemade and the soil moisture sensors were replaced with locally made products (ARP Co., Ltd., Hadano, Japan). This WD-3-WET-5Y TDR type soil water potential sensor can simultaneously measure volumetric water content (VWM), electric conductivity (EC) and temperature of the soil. Furthermore, this sensor is rated IP68; this rating means protection from contact with harmful dust and immersion in water with a depth of more than 1 m. Currently for the WSN, the soil water potential seems to be the most suitable measurement item whose aim is irrigation control [10]. For measuring the water potential in soil, it is necessary to measure it at different points in a field, because soil moisture varies even within the same field. Therefore, we also designed and developed a low-cost water potential sensor and connected it to the wireless sensor network. This sensor is produced experimentally to translate soil water content into voltage variation and is composed of a gypsum block and a simple electric circuit. Regarding the ground environment, the weather station and solar radiation sensor were replaced with a German-made weather station. The modified WSN in this study consists of a weather station, three soil moisture sensors and a soil water potential sensor.

Third, the data acquired from the WSN should be standardized in order to provide information services, especially when we integrate various data sources. Agricultural IoT is necessary for the next generation WSN and science-based agriculture so that the data obtained from the WSN are standardized and the standardized data are modified or combined to create indices that are related to the plant growth phases and useful for farmers. 

Last, the service provided in this study should be useful for farmers to cultivate high quality fruits. Along with this purpose, we developed two kinds of indices for cultivation; the first one is the “primary index” and the second one is the “secondary index”. The primary index is the monitored raw data that is made into a graph, or made into an at-a-glance list. The secondary index is the one that is made by modifying or combining the monitored raw data based on plant physiological theory. Therefore, the secondary index should be useful for farmers to improve their daily work. 

So far, some secondary indices are recognized, especially about the grapevine cultivation. In our web service, there are six indices; the Accumulated Growing Degree Days (AGDD) is the index for predicting the growing stage of fruit; the Growing Season Temperature (GST) is the index for deciding the species suitable in the temperature of the place; the Coolnight Index (CI) is the index that indicates how much secondary metabolite is contained in the wine grape; the Heliothermal Index (HI) is the index which uses the daily temperature and solar irradiance to evaluate the mass of photosynthetic products; the Biologically Effective Degree Days (BEDD) is the index which uses the daily temperature difference between highest and lowest in addition to the daily temperature as parameter to predict maturity of the wine grape; and the Dryness Index (DI) is the index which uses the temperature, relative humidity, precipitation, wind speed and solar duration as parameters to recognize the soil dryness state [11]. In addition to these indices, our service provides the solar duration data using the algorithmic program invented by Slob and Monna [12].

### 2.3. Diagnosis of Fruit Tree Vigor Using Optical Sensing

In the test field in the mandarin orange grove, a mulch and drip irrigation system was deployed four years ago. The Mulch sheet (Shibataya kakohshi Co., Ltd., Niigata, Japan) is a waterproof, moisture-vapor permeable sheet. Because of this Mulch sheet, most of the rainfall does not go into the soil so that meaningful irrigation control is possible. In addition, the growing condition was monitored by the WSN [3].

#### 2.3.1. Thermal Image Acquisition of Mandarin Orange Leaves

**Figure 1:**
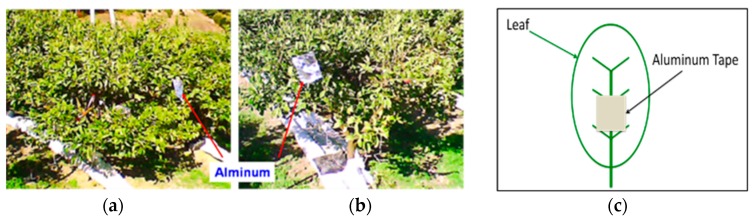
Aluminum as the control point in the field experiment: (**a**) Tree whose vigor strength is good; (**b**) Tree whose vigor strength is bad; (**c**) The usage of aluminum tape.

A series of laboratory scale experiments was carried out to determine the emissivity of mandarin orange leaves [5]. The thermal image of each mandarin orange leaf was taken by a Thermoshot thermography camera (Nippon Avionics Co., Ltd., Tokyo, Japan) from two trees with greatly different tree vigor under the same cultivation environment selected in the orange grove (Figure 1a,b). This thermography camera can measure from −20 to 100 °C and its resolution is 0.1 °C. The spectral range of this camera is from 8 to 13 μm, and its thermal image pixels are 160 × 120 pixels in size. The control area was marked on the thermal image using aluminum tape of which the emissivity greatly differed in order to facilitate the comparison between the visible and thermal images (Figure 1c).

**Figure 2:**
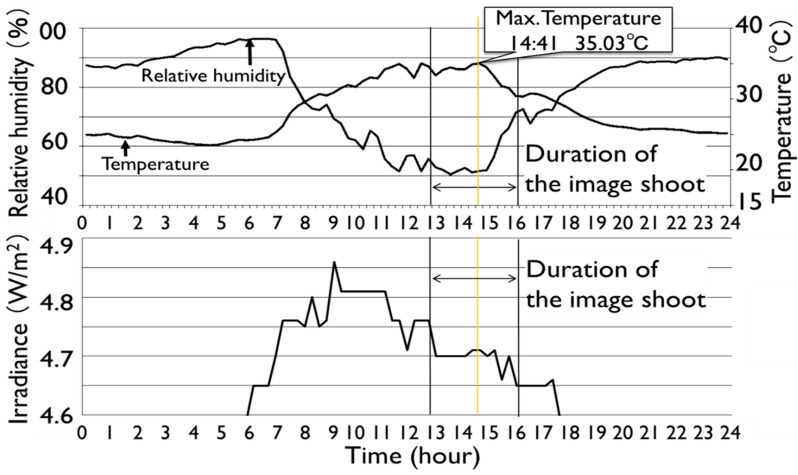
Cultivation condition of orange trees.

**Figure 3:**
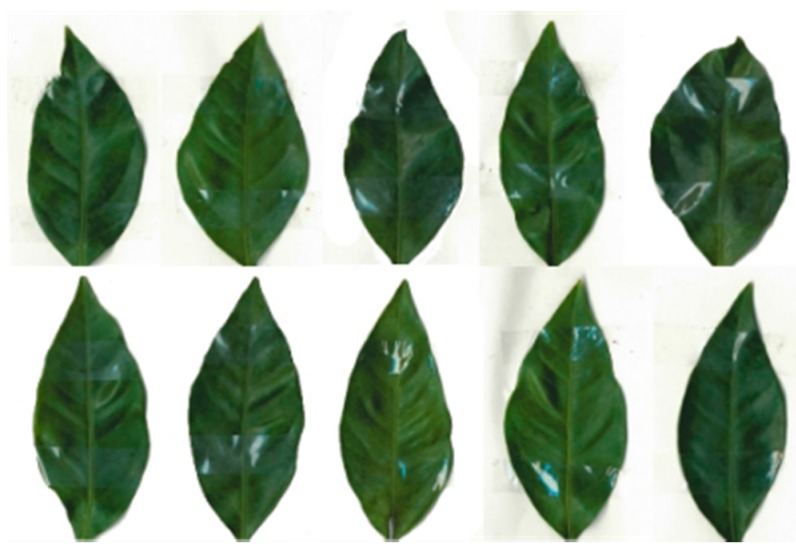
Sampled leaves used in this study.

The cultivation environment is shown in Figure 2. The emissivity determined in the laboratory (0.95) was applied in this experiment. A leaf template is needed to make a precise comparison of the temperature distribution between two leaves. Ten leaves with different shapes shown in Figure 3 were sampled in the orange grove and the average shape of the Satsuma Mandarin leaf was determined using the shape analytical method based on r-θ-φ coordinate system, which we have already developed [13]. This shape analysis was applied to the Satsuma Mandarin leaf.

Shape analysis was performed by the projection of a binary image of a leaf on tangent coordinate system. The origin was set on the center of gravity. A vector from the origin to a point on the orbital was auxiliary drawn as an arrow. Let r, θ and φ be the vector, angle of the vector and angle between the vector and tangent line at the point along the orbital, respectively. The orbital can then be projected into the tangent coordinate system with parameters θ and φ. Each obtained thermal image was then mapped to the template leaf and a comparison of temperature distribution was made between the strong leaf and weak one.

#### 2.3.2. Vigor Measurement of Mandarin Orange Leaves by Fluorescence and Fluorescence X-ray Methods

**Figure 4:**
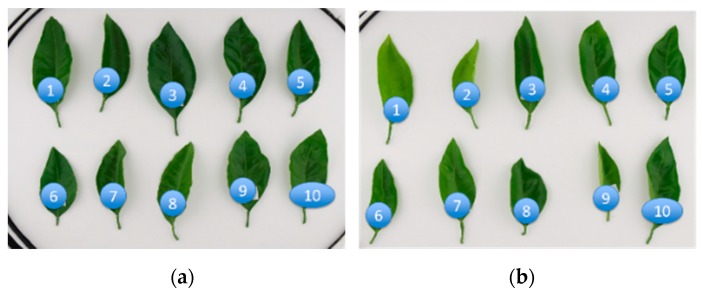
Sampled leaves: (**a**) Leaves of strong tree; (**b**) Leaves of weak tree.

Two types of leaves of which the vigor greatly differed in the same cultivation environment were selected from the orange grove. Ten leaves were taken from three weak vigor trees and another 10 leaves were taken from three trees with strong vigor (Figure 4). These leaves were taken from shoots without fruits. The evaluation criteria of tree vigor are based on the tree size, the number of leaves per tree, and the leaf color. The collected leaves were sent cool to the laboratory at Mie University using a courier and measurements by fluorescence and fluorescence X-ray methods were performed in the laboratory. As for the fluorescent measurement, a Dualex Scientific+ instrument (Force-A, Orsay, France) was used. It provided indices of the flavonols (FLAV), anthocyanins (ANTH), and chlorophyll (CHL) [14]. The leaf chlorophyll content was assessed by measuring the light transmission at 710 nm, absorbed by chlorophyll, and in the near-infrared (NIR) at 850 nm to take into account the effects of the leaf structure.

The chlorophyll Dualex index is given by the formula:

CHL = [(I_850_/I_0,850_)/(I_710_/I_0,710_)] − 1 (1)

where I and I_0_ are the signals measured with and without the leaf sample in the leaf clip, respectively. The Dualex (Dx) measures the leaf epidermal flavonols or anthocyanins at 375 and 520 nm, respectively, using the chlorophyll fluorescence (ChlF) screening method and equalizing the ChlF signals under these excitation wavelengths and that under a red excitation at 650 nm as a reference. 

Compounds present in the epidermis of the leaves attenuate the incident radiation before this can reach the first chlorophyll layer present in the mesophyll, depending on their absorption spectrum. Flavonols are the main flavonoids in dicotyledons absorbing UV radiation at 375 nm; therefore, the intensity of the ChlF induced by this radiation (ChlF_UV) will be inversely proportional to the epidermal flavonol concentration. Using a red light excitation, not attenuated by flavonols, a ChlF signal (ChlF_R) independent of the flavonol concentration is obtained. This signal is used as a reference. By comparing the ChlF signals from the two different excitations, the index of the flavonols can be calculated (in accordance with the Beer-Lambert law) as the logarithm of the ratio between the ChlF under red light and that under UV radiation:

FLAV = log(ChlF_R/ChlF_UV) (2)

The same concept applies to the determination of anthocyanins using a green light, absorbed by anthocyanins, instead of UV radiation. In addition to the above indices, the Dx sensor calculates the nitrogen balance index (NBI).

**Figure 5:**
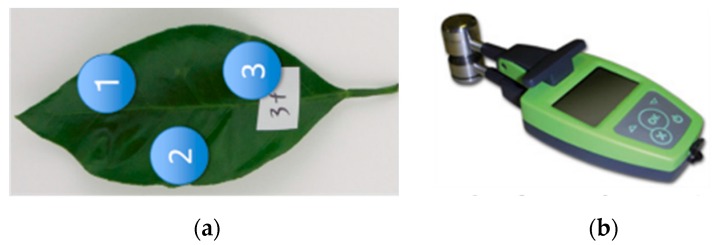
Fluorescent sensor used and measuring area of the leaf: (**a**) Fluorescent sensor used in this study; (**b**) Measurement areas in each leaf.

NBI = CHL/FLAV
(3)

as the ratio between the chlorophyll and flavonol indices that can be used as a proxy of the crop leaf nitrogen level. Fluorometric analysis with the measurements of the chlorophyll, flavonol and anthocyanin Dualex indices (CHL, FLAV, ANTH) was performed using 10 leaves of good color and another 10 leaves of bad color. The measurements were made at three areas of each leaf shown in Figure 5 in order to find the pigment distribution inside the leaf.

As for the fluorescence X-ray measurement, an Innov-X DELTA Premium Handheld XRF (hXRF) Analyzer (Olympus Corp., Tokyo, Japan) was used [15]. Although this hXRF sensor is portable, the specification of this sensor is as good as that of the Rayny EDX-700 (Shimadzu Corporation, Kyoto, Japan) for the laboratory use that was used in previous study [16]. Weighing roughly 2 kg, the instrument is equipped with a built-in camera/collimator mounted in the vicinity of the probe and a rechargeable Li-ion battery for easy field operation. We used the low beam energies of hXRF for the light elements, which are specifically those lighter than Mg since our target is the plant. In this study, although the hXRF analyzer was originally developed for use in the field, we mostly used the benchtop configuration of this analyzer. We used the same leaves shown in Figure 4 and analyzed them in the same way as the fluorescence method shown in Figure 5.

**Figure 6:**
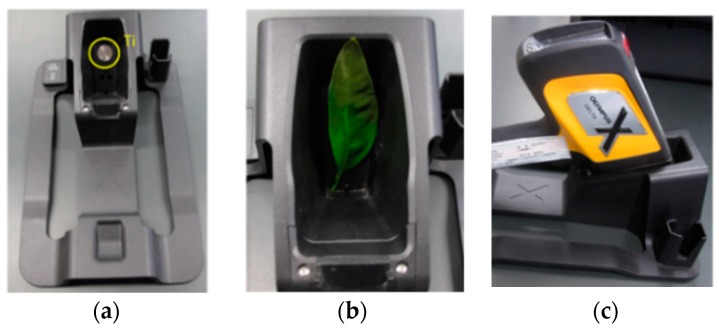
Special measurement station for leaf: (**a**) Titanium coin used instead of iron; (**b**) special measurement station for the leaves; (**c**) hXRF analyzer used in this study.

We monitored the internal instrument stability by measuring the Fe K-a counts on a 316 stainless steel coin every day using the Delta Docking Station (DDS). In addition, we made a special measurement station for the leaf with modifications that replace the iron coin to a titanium one because titanium is not present in the plant but is included in both the high and low beam energies (Figure 6a). Then, the leaf is set on the station and measured by the XRF sensor (Figure 6b,c)

XRF analysis was performed to 10 leaves of good color and another 10 leaves of bad color. The measurements were made at three areas of each leaf shown in the figure according to the built-in camera information in order to find the element distribution inside each leaf.

## 3. Results and Discussions

### 3.1. Growing Environment Measurement by the WSN

#### 3.1.1. Growing Environment Data Acquired by the WSN

**Figure 7:**
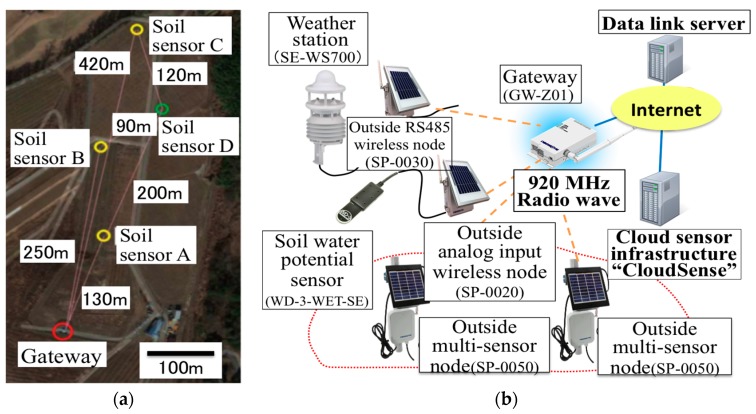
Structure of the WSN in Tomi-no-oka vineyard: (**a**) Installation points of sensors; (**b**) System configuration of WSN.

The new WSN system with the selected weather station (WS700-UMB, Lufft Inc., Berlin, Germany) and TDR type soil water potential sensors (WD-3-WET-SE, ARP Co., Ltd.) were designed and deployed in the vineyard. The weather station can acquire air temperature, relative humidity, air pressure, wind speed, wind direction, 1 min precipitation and solar irradiance data, while the soil water potential sensor can acquire the soil volumetric water content, soil temperature and soil electrical conductivity. These data are collected by gateway via 920 MHz radio and sent to the data link server that is connected to the gateway (Figure 7).

To send these acquired data to the cloud sensor infrastructure, the data link server uses HTTP-GET API defined by the cloud sensor infrastructure. In the revised WSN system, we use the SPPNet protocol produced by Sumitomo Precision Products Co., Ltd. The weather station is connected to the sensor node (SP-0030) by a two-wire RS485 network. Connecting the node to the RS485 serial interface devices can make a 1 to 1 or 1 to *N* see Duplex wireless communication system.

The power source is the AC 100 V commercial power supply existing in the vineyard which is converted to DC 24 V. On the other hand, each soil moisture sensor is connected to the analog sensor node (SP-0020) with specifications similar to those of the weather station. Its electricity source is DC from a solar panel deployed above the soil moisture sensor. These sensor nodes were developed in this study in order to connect the sensor to this WSN system (Table 1). The data link server of this WSN system has a customized web application based on SPP’s commercial product ‘EcoWizard’ which has HTTP Server capability, DB and Flash Application.

**Figure 8:**
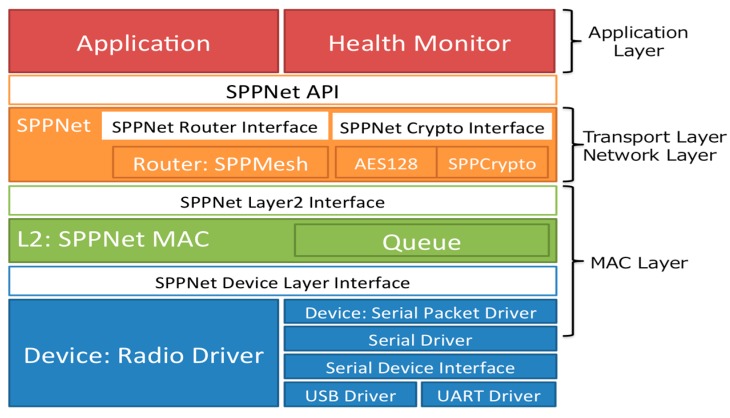
The information about SPPNet protocol used in this study.

Regarding the data communication, the Modbus protocol is applied and the frequency of the radio used in this WSN system is a 920 MHz radio wave based on the Wireless Smart Utility Network which enables long-range wireless communication. Thanks to the 920 MHz, wave attenuation caused by the plant is not significant compared to the 2.4 GHz used in the previous WSN. In addition, this SPPNet protocol can provide the wireless transmission system of sensors compatible with Modbus protocol easier than in the eKo sensor network. All environmental data collected by the sensors in the WSN are sent to the Modbus master (Gateway GW-Z01), then stored in the data link server. The Over-The-Air throughput of this network is more than 100 kbps in the ideal state, while the actual throughput is more than 24 kbps. The MAC of this network is CSMA/CA system and comply with the transmission time restrictions defined in Association of Radio Industries and Business in Japan (ARIB) STD-T108. The routing protocol of this system ad-hoc mesh networking protocol, self-organizing and selt-healing. In addition, Transmission latency of this system is from 2 to 912 ms/hop. Other information about this system is shown in Figure 8. 

**Figure 9:**
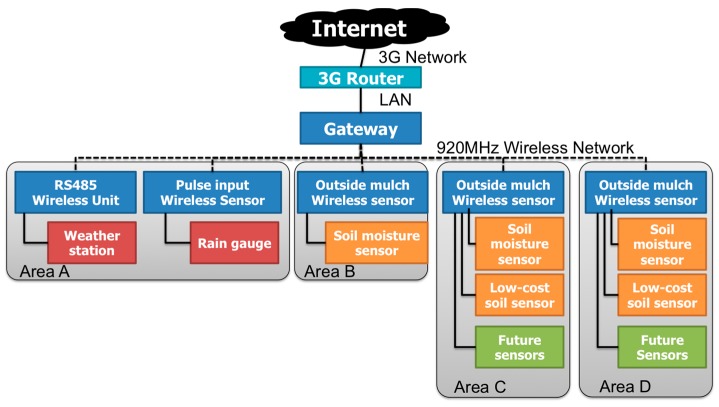
The structure of the WSN used in this study.

This WSN consists of a weather station and three soil moisture sensors and a soil water potential sensor (Table 2, Figure 9). The soil water potential sensor was manufactured for this study to translate the soil water content into a voltage variation, and composed of a gypsum block and simple electric circuit to reduce material costs. As a result, it costs only about 5000 yen to produce this sensor and the process is so simple that farmers can do it by themselves. As the sensor for measuring the other soil information, the WD3-WET-SE soil moisture sensor was selected. It can measure the soil volumetric moisture content, soil electric conductivity and soil temperature at the same time. In addition, this sensor is worthy of the IP68 code that means it is completely resistant to both water and dust. Thus, we will be able to use this sensor in the ground for a long period. As the weather station, a SE-WS700 system was selected. This weather station can acquire the data of air temperature, relative humidity, air pressure, wind speed, wind direction, 1 min_precipitation and solar irradiance at the same time, which enables a cheaper WSN system than in the previous work [3]. In addition, this weather station can measure each environmental data point every one minute. The important point is the accuracy of each measurement acquired by this weather station, especially the wind speed measured by the ultrasonic anemometer and precipitation measured by the Doppler radar rain gauge. Furthermore, its water sampler requires minimal maintenance [17]. Thus, this weather station has the potential to solve the problem raised in the previous work.

In order to provide information services, especially when we integrate various data sources, interoperability of the data and system is important. Therefore, we used the Sensor Observation Service (SOS) to standardize the terminology and Units of Measure (UOM) (Table 3).

SOS is a web service to query real-time sensor data and sensor data time series (http://www.opengeospatial.org/standards/sos). This service applies XML encoding for observation & measurement (O & M) data originating from the sensors. It defines a web service interface that allows querying observations, sensor metadata, as well as representations of observed features

Following the OGC standard, defines the means to register new sensors, to remove existing ones and to insert new sensor observations. The cloud sensor infrastructure called “cloudSense” has a sensor backend service based on the SOS, thus making it possible for us to add and modify sensors and automatically reflect the change in the sensor configuration without program modification [18,19].

**Figure 10:**
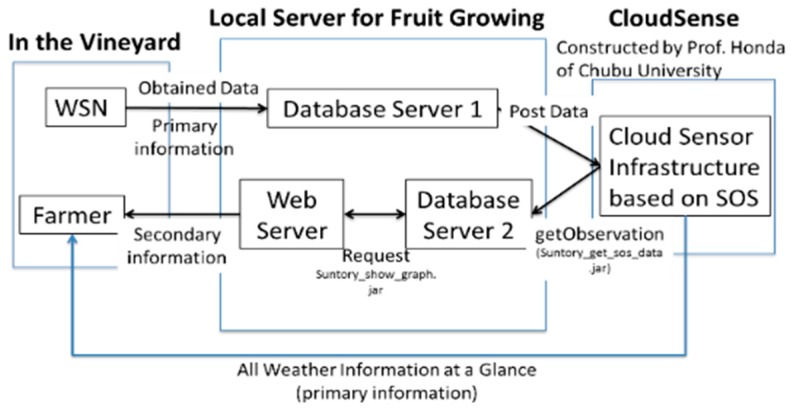
Design of agricultural IoT as a service for farmers

In order to send these environmental data from the data link server to the cloudSense, the data link server has HTTP-GET API defined by SOS. The terminology, physical quantity and UOM of each observed data point are standardized and stored in the database in cloudSense (Figure 10).

Figure 10 shows the data flow of the IoT service in this study. The obtained data goes to the local server for WSN. The dataset then goes to the cloudSence for standardization. The standardized data are back to the Database of the local server. The primary information and the secondary information service were then delivered to the farmers at the field. 

**Figure 11:**
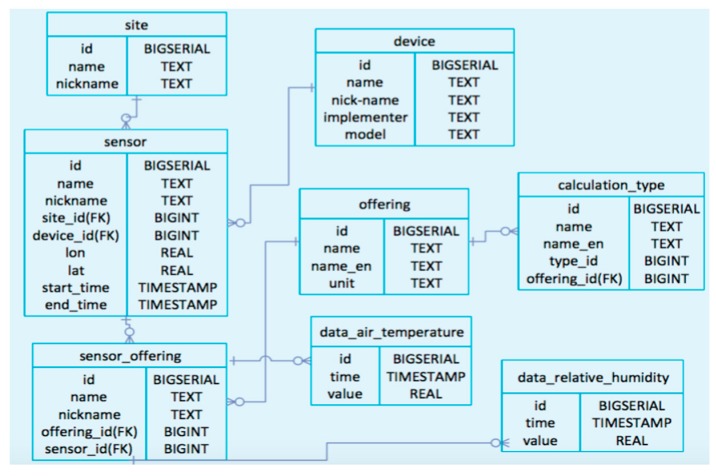
ER chart of database structure.

The standardized data stored in cloudSense are also acquired using HTTP-GET API defined by SOS. In the HTTP-GET API, there are several kinds of requesting methods. First, “getCapability” is the method that can confirm the information about each observed data. Secondly, “getObservation” is the method that can acquire the monitored value of the selected observed property. When you use the getObservation request, you should set “eventTime”, that sets both the start time and end time, and “observedProperty” which decides what sensor to choose, then one can obtain the monitored data described by the O & M language based on XML. Using these methods, the standardized data are sent to the server located at the Mie University, then XML tags are parsed and the extracted data are stored in the database constructed in this study (Figure 11). 

Verifying the existence and accuracy of the weather data obtained from the weather station is necessary. Therefore, we used the “getObservation” method of cloudSense to make sure whether this system can acquire information about the growing environment from the WSN and the connection between the data link server and cloudSense, then we could confirm that the data obtained from the WSN are stored in the cloudSense. Concerning the values from the soil moisture sensor, there are some deficit points. In our web application, each missing value is replaced with an average value of the value former day and the value of the following day. In addition, we also compare the data from the SE-WS700 weather station and the data from the reference weather station. The reference weather station is added to the WSN and deployed next to the SE-WS700. As a result, almost all the observed data were correct, except for the precipitation and air temperature data [20,21].

#### 3.1.2. IoT service for Farmers

**Figure 12:**
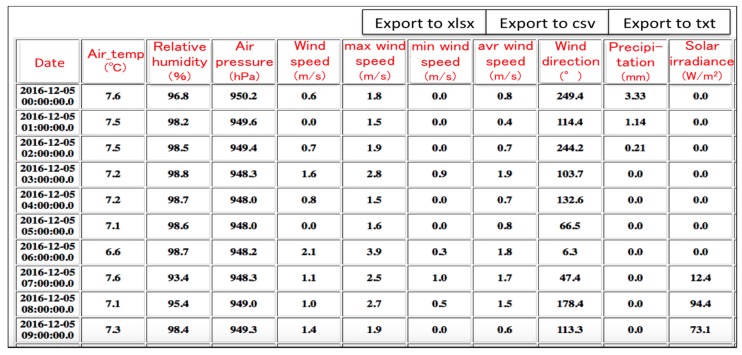
Primary index as a list displayed in web service.

By getting the standardized data from the cloudSense and storing them in our server, we can create a web service that offers various kinds of cultivation indices to the farmers in the field. In this web site, growing environment information, as a primary index, is displayed as a list of numeric values (Figure 12). All the data acquired by the weather station are simultaneously displayed every one hour. 

**Figure 13:**
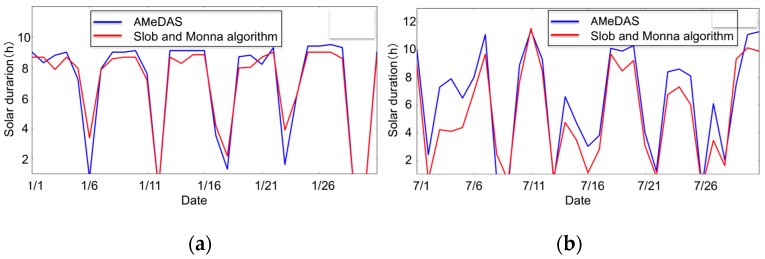
Data verification about solar duration: (**a**) The data acquired in January; (**b**) The data acquired in July.

In addition to the primary index, our web service also provides several secondary indices; AGDD, GST, CI, HI, BEDD, and DI. They are calculated using growing environment data standardized by cloudSense. The validation of the solar duration data that is necessary for the DI calculation validity was made to verify the certainty of this algorithmic program for calculating the solar duration by comparing it with the true value obtained from the solar duration sensor next to the weather station. Figure 13 shows the result of the solar duration data verification. When comparing in the winter, the difference between the true value and the value obtained from the algorithmic program is only 3 to 5%, while when comparing in the summer, the difference between these two values is about 10%. Based on this result, we should modify the solar duration data used in this study. 

**Figure 14:**
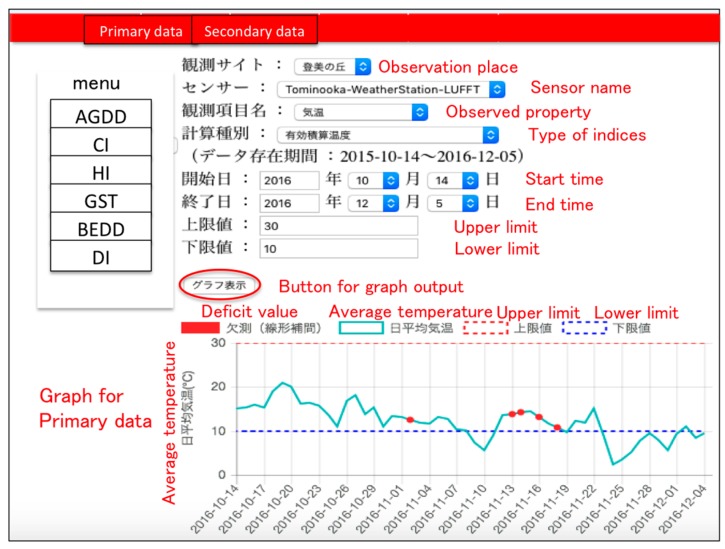
Secondary index displayed in web application. Since this application is written in Japanese, English annotation is mentioned with red letter.

**Figure 15:**
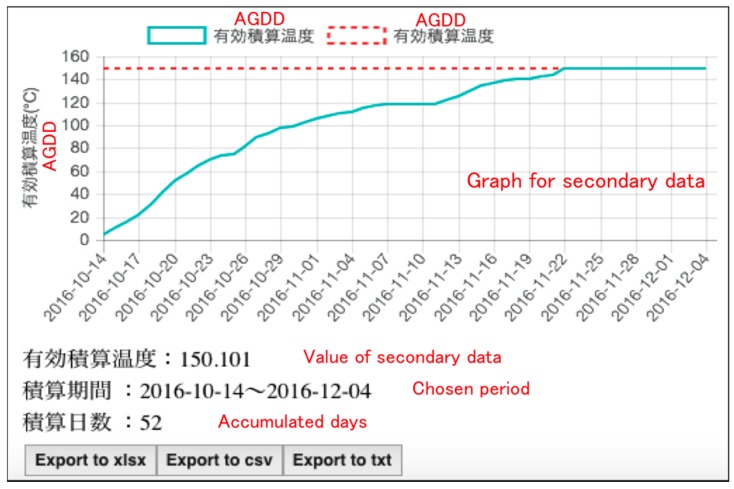
Polygonal line graph shown in this web application.

In the service for secondary indices shown in Figure 14 and Figure 15, users can select the observation place, type of sensor, observed property, type of index and time period that you want to observe (Figure 14). After they choose, the result of both the chosen secondary index and primary index is graphically shown (Figure 15). Figure 15 shows the result of the chosen primary and secondary indices in the web application developed in this study. In order to verify the accuracy of each secondary index; AGDD, GST, CI, HI, BEDD, DI, we compared each data point about the CI and HI obtained from this service and corresponding data obtained from previous research, then the validity of the secondary index calculated by our service is proved [22].

#### 3.1.3. Vigor Sensing of Mandarin Orange Leaves Using Thermal Image

**Figure 16:**
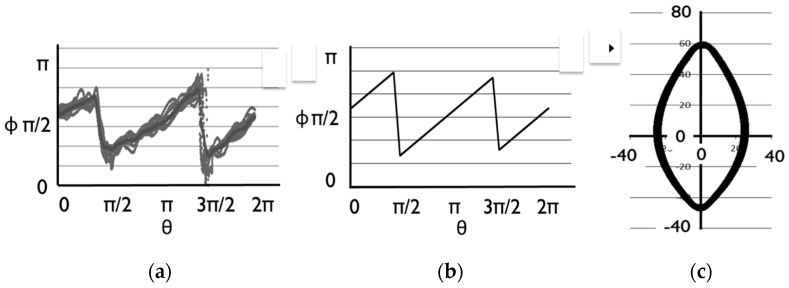
Transformation of leaves: (**a**) Transformed data; (**b**) Average of bundle; (**c**) Inverse transformation.

The results of the transformation are shown in Figure 16. The bundle of Figure 16a corresponds to the shapes of ten leaves and the line in the bundle indicates the average. The line shown in Figure 16b was adopted as a template shape because a symmetrical shape would be better for easy analysis. A reverse transformation was made for the line in Figure 16b and the template shape shown in Figure 16c was derived.

**Figure 17:**
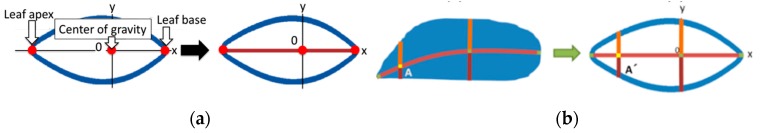
Mapping of the leaf to the template: (**a**) Sample of the mapping; (**b**) apply this mapping method to real leaf.

The coordinate system was defined for the original shape as well as the template shape, which was explained by using the pattern diagram shown in Figure 17. Figure 17a shows the determination method of the coordinates for the template while Figure 17b is for the original shape. For both, the center of gravity corresponds to the origin. The *X*-axis corresponds to the straight line via the leaf apex center of gravity and leaf base for the template image while the parabola is for the original image. The *Y*-axis is perpendicular to the *X*-axis at the center of gravity. Each pixel of the original image was transformed in manner similar to Figure 17b.

**Figure 18:**
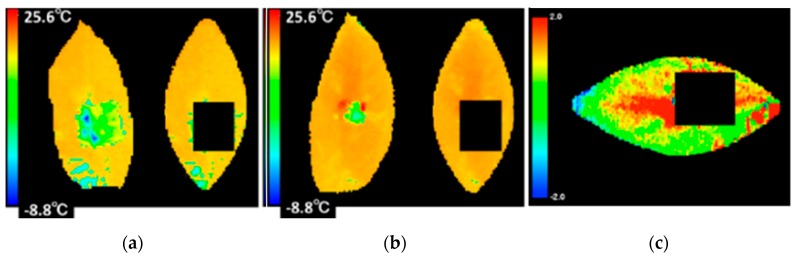
Thermal images of the leaves: (**a**) Strong leaf; (**b**) Weak leaf; (**c**) Temperature difference between strong leaf and weak one.

**Figure 19:**
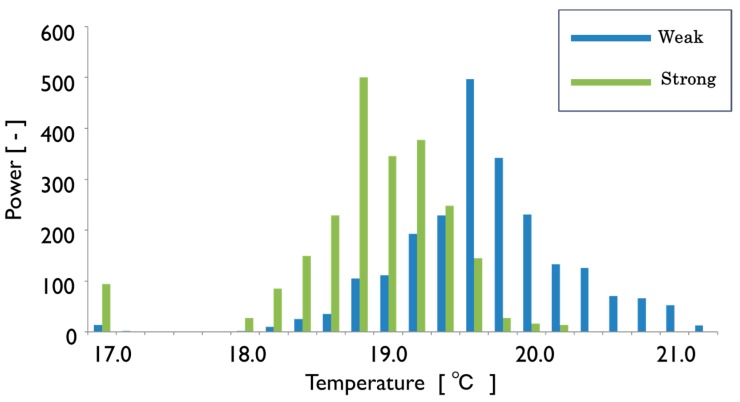
Comparison of temperature distribution between weak leaf and strong one.

Figure 18 show the two sets of the original image and transformed one for the strong tree and the weak one. The thermal image in Figure 18a has the temperature distribution lower than that in Figure 18b, which coincides with the fact that the strong tree has a larger amount of transpiration than the weak tree. It can be found that the normalized shape can produce the distribution of temperature difference within the leaf surface as is shown in Figure 18c and Figure 19.

#### 3.1.4. Vigor Measurement of Mandarin Orange Leaves by Using Fluorescent Method

**Figure 20:**
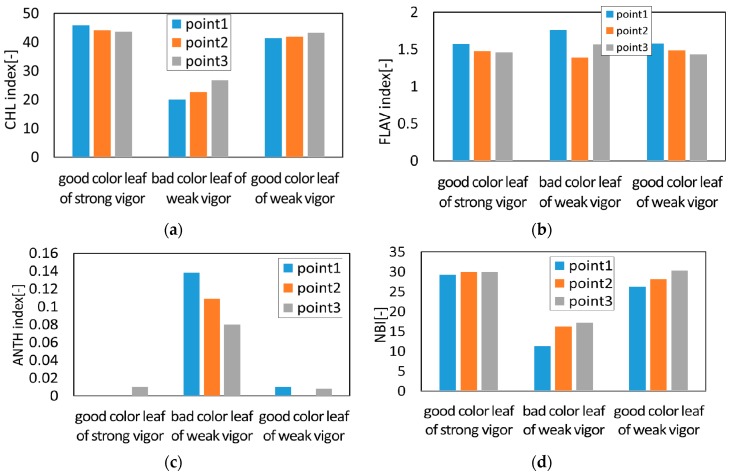
Comparison of each index of various leaves: (**a**) The rusult of chlorophyll; (**b**) The result of flavonol; (**c**) The result of anthocyanin; (**d**) The result of NBI.

The measurements were made at three areas in each leaf shown in the Figure 5b in order to find the pigment distribution inside the leaf. First, the distribution of pigments inside each leaf was examined for 10 leaves taken from three trees of weak vigor (weak leaf) and for another 10 leaves taken from three trees of strong vigor (strong leaf). Each bar in the chart of Figure 20 shows the difference among the three areas for the good color leaf of strong vigor, bad color leaf of weak vigor and good color of weak vigor. Figure 20a corresponds to the result of chlorophyll (CHL), Figure 20b corresponds to that of flavonol (FLAV), Figure 20c corresponds to that of anthocyanin (ANTH), and Figure 20d corresponds to that of nitrogen balance index (NBI). 

As a result, the difference in each value among the three areas was hardly found in the case of the strong leaf. Meanwhile, as is shown in Figure 20, in the case of the weak leaves, it was found that a leaf with a good color has a similar tendency as the strong leaf, and the leaf with bad color has a difference among the three areas inside the leaf for CHL and NBI. Although there is a little problem in the weak leaf, the average value of the three areas was used as a representative value of each leaf in the later discussion. According to the results of the CHL, FLAV, ANTH and NBI for 10 weak leaves and for another 10 strong leaves, the CHL is distributed from 22 to 42 for the weak leaves, while it is distributed from 38 to 52 for the strong leaves. The ANTH is around 0.1 for the weak leaves and the existence of anthocyanin was confirmed while it was almost 0 in the strong leaves and no anthocyanin was found. The FLAV is distributed from 1.4 to 1.8 for the weak leaves while it is distributed from 1.3 to 1.6 for the strong ones. The tendency that the FLAV of the weak leaf is slightly higher than that of the strong leaf was observed. The NBI showed a similar tendency to the CHL. That is, the NBI is distributed from 13 to 30 for the weak leaves while it is distributed from 30 to 42 for the strong ones. 

**Figure 21:**
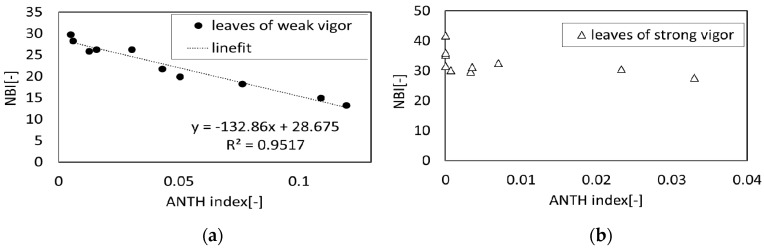
Correlation between ANTH and NBI: (**a**) Leaves of strong vigor; (**b**) Leaves of weak vigor.

Figure 21 shows the relationship between ANTH and NBI for strong leaves and weak leaves. As for the strong leaves, a weak negative correlation was found between the ANTH and NBI (Figure 21a). Meanwhile, in the case of the weak leaf, a strong negative correlation was found and the correlation coefficient was 0.95 (Figure 21b).

Generally, photosynthesis is caused by a light reaction (electron transfer system) that generates biochemical energy such as ATP and NADPH and a dark reaction (Calvin cycle) that immobilizes the carbon dioxide using these biochemical energies. The energy of sunlight is entirely used to immobilize the carbon dioxide when the light reaction is balanced with the dark reaction. However, the surplus energy that is not used for immobilization of the carbon dioxide contributes to active oxygen generation in the plant body and tissue destruction occurs when the solar energy uptake by the light reaction exceeds the carbon dioxide fixation by the dark reaction. As one of the defense mechanisms to this phenomenon, it is known that resolving the chlorophyll that takes part in the light reaction and generating anthocyanins reduces the surplus sunlight, and the light reaction is made to balance with the dark reaction [23]. The negative correlation between the ANTH and CHL obtained in our experiments seems to be consistent with this fact.

#### 3.1.5. Vigor Measurement of Mandarin Orange Leaves Using Fluorescent X-ray Method

It is necessary to determine the suitable beam exposure time for the fluorescence X-ray analysis of a leaf. Firstly, after changing the irradiation time to 30, 60, 90, 120 and 150 s we carried out FXR analysis of the leaf, using two kinds of beams, beam 1 and beam 2. As a result, the characteristic peak heights for the typical elements were almost the same in all the data. Therefore, the irradiation time for beam 1 was selected to be 30 s, and that for beam 2, as 60 s and these irradiation times were used for all the experiments. The measured values of the fluorescence X-rays were normalized using the peak intensity of RhLα (approximately 2.71 keV) generated by irradiation [8]. The measurements were then made at three areas of each leaf shown in the Figure 5b in order to find the element distribution inside the leaf. The distribution of elements inside each leaf was examined for 10 leaves taken from three trees of weak vigor (weak leaf) and for another 10 leaves taken from three trees of strong vigor (strong leaf) by fluorescence X-ray analysis the same as the fluorometric analysis. 

**Figure 22:**
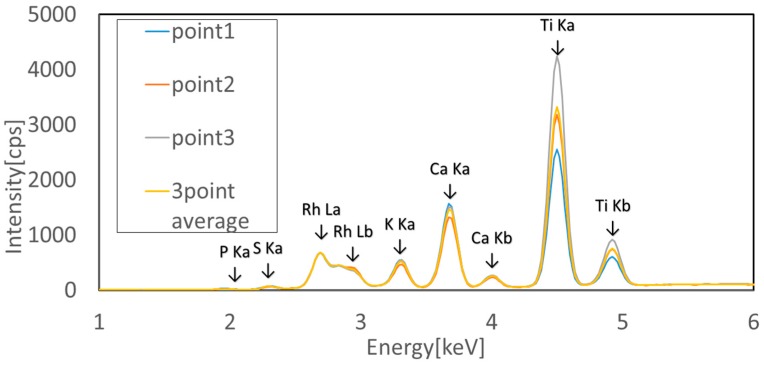
Normalised fluorescence X-ray spectroscopic data of strong leaf.

As a result, both the weak and strong leaves have a slight difference in the element contents among the three areas. Figure 22 shows the FXR spectroscopic data of three areas for the strong leaf. Although there is a slight distribution inside the leaf, the average value of the three areas was used as a representative value of each leaf in the later discussion. Table 4 shows the average element values of 10 weak leaves and that of 10 strong values. The kind of the detected element was quite different between these two groups. Twelve kinds of elements were detected in the weak leaf while 24 kinds were found in the strong leaf.

The meaning of the amount of each detected element for the tree vigor was then examined using “The Shizuoka Prefecture Soil Fertilizer Handbook” as a reference (Table 5). 

Table 5 shows the information on the appropriate kinds and contents of the elements inside the mandarin orange leaf. A comparison was made between the actual measurement results in Table 4 and a desirable amount of the element listed in Table 5. As a result, we had a diagnostic outcome that the content of Ca, K, Fe, Zn, and Cu are suitable, P is a somewhat small amount, and Ni and Mo are in excess for the strong leaf, while Ca and K are suitable, Fe is in a somewhat small amount, P is lacking, and Mn and Ni are in excess for the weak leaf (Table 4). In this diagnosis, however, the elements of N and B cannot be detected by the hXRF among the essential elements shown in Table 5, and the proper quantity of Mg for the plant, 0.3 to 0.6%, was not evaluated because the hXRF techniquer can only detect weight percent concentrations of Mg of no less than 1% in a sample.

### 3.2. Future Prospects of the Cultivation Indices and Tree Vigor Sensing

In this study, we developed the cultivation management system that provides cultivation indices for farmers at the vineyard. On the other hand, we also verified the effectiveness of mandarin orange tree vigor diagnosis using multi-band optical sensing. 

The next objective of our study has four topics: firstly, to confirm the availability of the cultivation indices for grapevine in Japan, secondly, to replace the WSN in the orange grove and introduce a cultivation management system similar to the system in the vineyard and to develop appropriate cultivation indices for mandarin orange in Japan, thirdly, to verify the availability of the tree vigor diagnosis using multi-band optical sensing of vines and lastly, to store multi-band optical sensing data related to tree vigor in cloudSense and to standardize the terminology and UOM.

Since the main aim of this kind of service is to improve farmers’ cultivation management practices, growing environmental information and tree vigor information must be combined from the plant physiological point of view in order to provide more useful cultivation indices. Recently, several studies about sap flow sensing and translocation in the phloem have been started. Especially, sap flow sensing research has been carried out in the Tomi-no-oka vineyard and we plan to connect a sap flow sensor to the SP-0050 node in the WSN [24]. On the other hand, research on the sensing of translocation in the phloem by using IR-spectroscopy is also ongoing [25,26]. This information could play an important role to improve cultivation indices.

## 4. Conclusions

We have developed a new WSN with a weather station and soil moisture sensors. The integrated system consisting of the WSN system and cloudSense will enable us to develop an effective web application for farmers. In future studies, there are three tasks, such as improvement of the durability of the WSN and accuracy of the sensors, a web application service that shows indexes of plant growth useful for farmers and a suitable cloud design for agricultural service, and design of a low-cost and energy-saving WSN.

The results in this paper could play a very important role in the health diagnosis of plants for agriculture based on optically sensed results. As for the optical and spectroscopic sensing, the combination of some sensing techniques could extract more precious quality information. It is necessary to totally understand the integrative quality of agricultural products, and the optically sensed information should be represented in the information to be easily understood, and the development of visualization technology for the complex sensing information of agricultural products is also desired. 

In the near future, the advanced sensing technology such as sap flow sensing could be linked to WSN and the physiologically-based knowledge of agricultural plants. By the assimilation and optimization of such subjects, the comprehensive vigor of agricultural plants could be evaluated by the science-based system for the sustainable supply of high quality agricultural materials. Furthermore, multi-band optical sensing data relating with tree vigor should be stored in cloudSense in order to provide more useful cultivation indices for farmers at the field. We shall also apply WSN knowledge acquired in vineyard to the mandarin orange grove and also apply the multi-band optical sensing knowledge acquired in mandarin orange grove to the vineyard.

## Figures and Tables

**Table 1:** WSN Devices.

Product Name	Description	Sensor Interface	RF	Power	Battery Life w/o Sunlight	Waterproof
SP-0020	Used for sensing analog sensors; Prototyping Model for PoC	Arduino UNO, which has analog (0–5 V) inputs	SPP’s original 920 MHz RF module	Solar Panel (2.15 W) Rechargeable batteries (9600 mAh)	<3 days	IP65
SP-0030	Used for sensing RS485 sensors; Prototyping Model for PoC	SPP’s original RS485 interface board		Solar Panel (2.15 W) Rechargeable batteries (9600 mAh)	<3 days	IP65
SP-0050	Used for sensing analog, digital sensors; SPP’s Commercial Model, developed based on SP-0020	SPP’s original board which has multiple interfaces such as analog (0–5 V, 4–20 mA), digital inputs		Solar Panel (1.4 W) Rechargeable batteries (3200 mAh)	<10 days *Low power mode	IP66
GW-Z01	Used for connecting WSN to the internet; SPP’s Commercial Model	N/A		AC Adaptor 5 V/1.6 A	N/A	N/A Deployed in Waterproof Box

**Table 2:** Sensor information used in this study.

Sensor Name	Model Number	Note
Weather station	WS700-UMB	Adoption of ultrasonic anemometer. Adoption of doppler radar rain gauge. Free from maintenance.
Soil moisture sensor	WD-3-WET-SE	Being able to measure EC (0–1 V), VWC (0–1 V) and temperature (0–1.2 V) of soil. Worthy of IP68 code.

**Table 3:** Sensor name, physical quantity and UOM.

Sensor Name	Physical Quantity	UOM
air_temperature	Temperature	Cel
air_pressure	Air pressure	hPa
wind_speed	Wind speed	m/s
10 min_maximum_wind_speed	Wind speed	m/s
10 min_minimum_wind_speed	Wind speed	m/s
10 min_average_wind_speed	Wind speed	m/s
wind_direction	Aind direction	deg
1 min_precipitation	Precipitation	mm
solar_irradiance	Global radiation	W/m^2^
10 min_maximum_solar_irradiance	Global radiation	W/m^2^
10 min_minimum_solar_irradiance	Global radiation	W/m^2^
10 min_average_solar_irradiance	Global radiation	W/m^2^
relative_humidity	humidity	%

**Table 4:** Average element values of strong and weak values.

Metal	Average Content (%, ppm)	Sample Number	Evaluation	Metal	Average Content (%, ppm)	Sample Number	Evaluation
LE (%)	73.7	30		LE (%)	71.9	30	
Ti (%)	20.3	30		Ti (%)	22.4	30	
Ca (%)	3.30	29	Optimum	Ca (%)	3.19	30	Optimum
K (%)	1.33	29	Optimum	K (%)	1.01	30	Optimum
S (%)	0.230	29		S (%)	0.201	30	
P (%)	0.112	26	A little deficiency	P (%)	0.0952	30	Deficiency
Si (ppm)	855	29		Si (ppm)	764	30	
Mn (ppm)	380	30	Excess	Mn (ppm)	375	30	Excess
Fe (ppm)	138	30	Optimum	Fe (ppm)	155	30	A little excess
Cu (ppm)	87.2	30	A little excess	Cu (ppm)	72.0	19	A little excess
Zn (ppm)	49.5	14	Optimum	Zn (ppm)	37.4	8	Optimum
Mo (ppm)	22.3	22	Excess	Mo (ppm)	23.5	19	Excess
Ni (ppm)	20.2	6	A little excess	Ni (ppm)	42.7	4	Excess
Mg		0		Mg		0	
Co		0		Co		0	

**Table 5:** Information on the appropriate kinds and contents of elements inside a leaf.

**Crop Name**	**Content**	**Concentration in Dry Matter (%)**										
		**Nitrogen (N)**		**Phosphorus (P)**			**Potassium (K)**		**Calcium (Ca)**		**Magnesium (Mg)**	
mandarin orange	deficiency	under 2.3		under 0.10			under 0.7		under 2.0		under 0.10	
	optimum	2.9~3.4		0.16~0.20			1.0~1.6		3.0~6.0		0.30~0.60	
	excess	over 4.0		-			over 1.8		over 7.0		-	
**Crop Name**	**Content**	**Concentration in Dry Matter (ppm)**										
		**Boron (B)**	**Manganese (Mn)**		**Iron (Fe)**	**Zinc (Zn)**		**Copper (Cu)**	**Molybdenum (Mo)**	**Nickel (Ni)**		**Cobalt (Co)**
mandarin orange	deficiency	under 30	under 30		under 35	under 10		under 4	under 0.05	-		-
	optimum	30~100	30~100		50~150	30~100		10~50	0.2~3.0	2.0~15		5~20
	excess	over 170	over 150		over 250	over 200		over 150	-	over 25		over 30
